# In Vitro Antimalarial Activity of Trichothecenes against Liver and Blood Stages of *Plasmodium* Species

**DOI:** 10.1021/acs.jnatprod.3c01019

**Published:** 2024-01-23

**Authors:** Prakash T. Parvatkar, Steven P. Maher, Yingzhao Zhao, Caitlin A. Cooper, Sagan T. de Castro, Julie Péneau, Amélie Vantaux, Benoît Witkowski, Dennis E. Kyle, Roman Manetsch

**Affiliations:** † Department of Chemistry and Chemical Biology, Northeastern University, Boston, Massachusetts 02115, United States; ‡ Center for Tropical and Emerging Global Diseases, University of Georgia, Athens, Georgia 30602, United States; § Malaria Molecular Epidemiology Unit, Institut Pasteur du Cambodge, 5 Boulevard Monivong, PO Box 983, Phnom Penh, 120 210, Cambodia; ∥ Department of Pharmaceutical Sciences, Northeastern University, Boston, Massachusetts 02115, United States; ⊥ Center for Drug Discovery, Northeastern University, Boston, Massachusetts 02115, United States; # Barnett Institute of Chemical and Biological Analysis, Northeastern University, Boston, Massachusetts 02115, United States

## Abstract

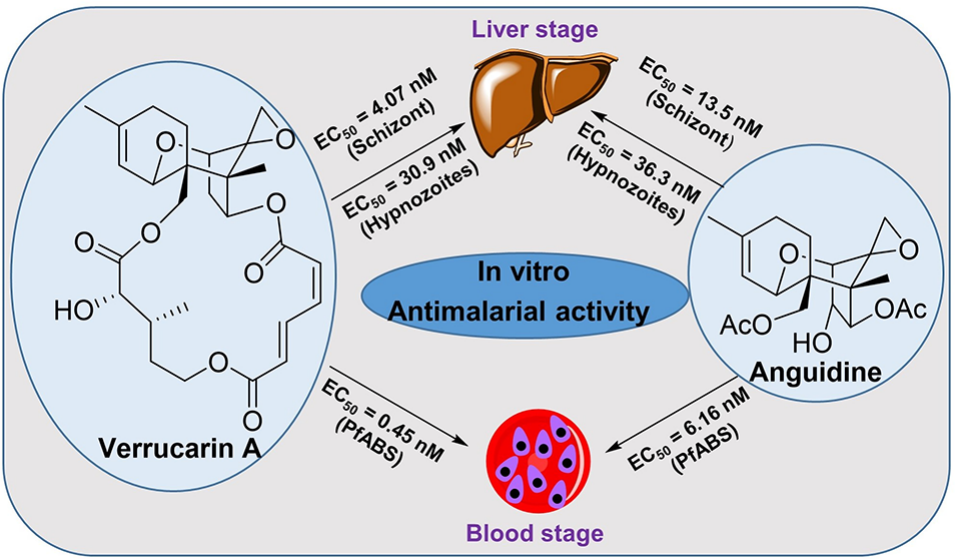

Trichothecenes (TCNs) are a large group of tricyclic sesquiterpenoid mycotoxins that have intriguing structural features and remarkable biological activities. Herein, we focused on three TCNs (anguidine, verrucarin A, and verrucarol) and their ability to target both the blood and liver stages of *Plasmodium* species, the parasite responsible for malaria. Anguidine and verrucarin A were found to be highly effective against the blood and liver stages of malaria, while verrucarol had no effect at the highest concentration tested. However, these compounds were also found to be cytotoxic and, thus, not selective, making them unsuitable for drug development. Nonetheless, they could be useful as chemical probes for protein synthesis inhibitors due to their direct impact on parasite synthesis processes.

Malaria is a serious infectious disease that affects almost half of the world’s population, with over 200 million cases reported annually.^1−3^ According to the WHO, there were approximately 249 million reported cases of malaria in 85 countries in 2022. After decades of year-over-year declines, there was an increase of 11 million cases between 2019 and 2020, which has been attributed to suspended control efforts due to the COVID-19 pandemic and international conflicts.^3^ Malaria is caused by one of five species of protozoan parasites in the *Plasmodium* genus (*P. falciparum*, *P. vivax*, *P. ovale*, *P. malariae*, and *P. knowlesi*), with *P. falciparum* being the most common and deadly globally, particularly in sub-Saharan Africa. *P. vivax* is the second most common species, found in Central and South America, as well as regions of Africa and Asia.^3−5^

Human malaria infection begins with an asymptomatic liver stage, followed by a cyclic symptomatic blood stage, as demonstrated in the *Plasmodium* spp. life cycle (Figure 1).^6−8^ These parasites have multiple developmental stages that allow them to infect and move between their mosquito and human hosts. When an infected female *Anopheles* mosquito bites a human, *Plasmodium* sporozoites leave the salivary glands, enter the host circulation, and quickly move to the liver for one round of asexual replication before breaking out and infecting the blood. For *P. vivax* and *P. ovale*, some sporozoites enter the liver and develop into a uninucleated hypnozoite, which remains dormant for months or years before reactivating and causing a relapse infection.^9^

**Figure 1:**
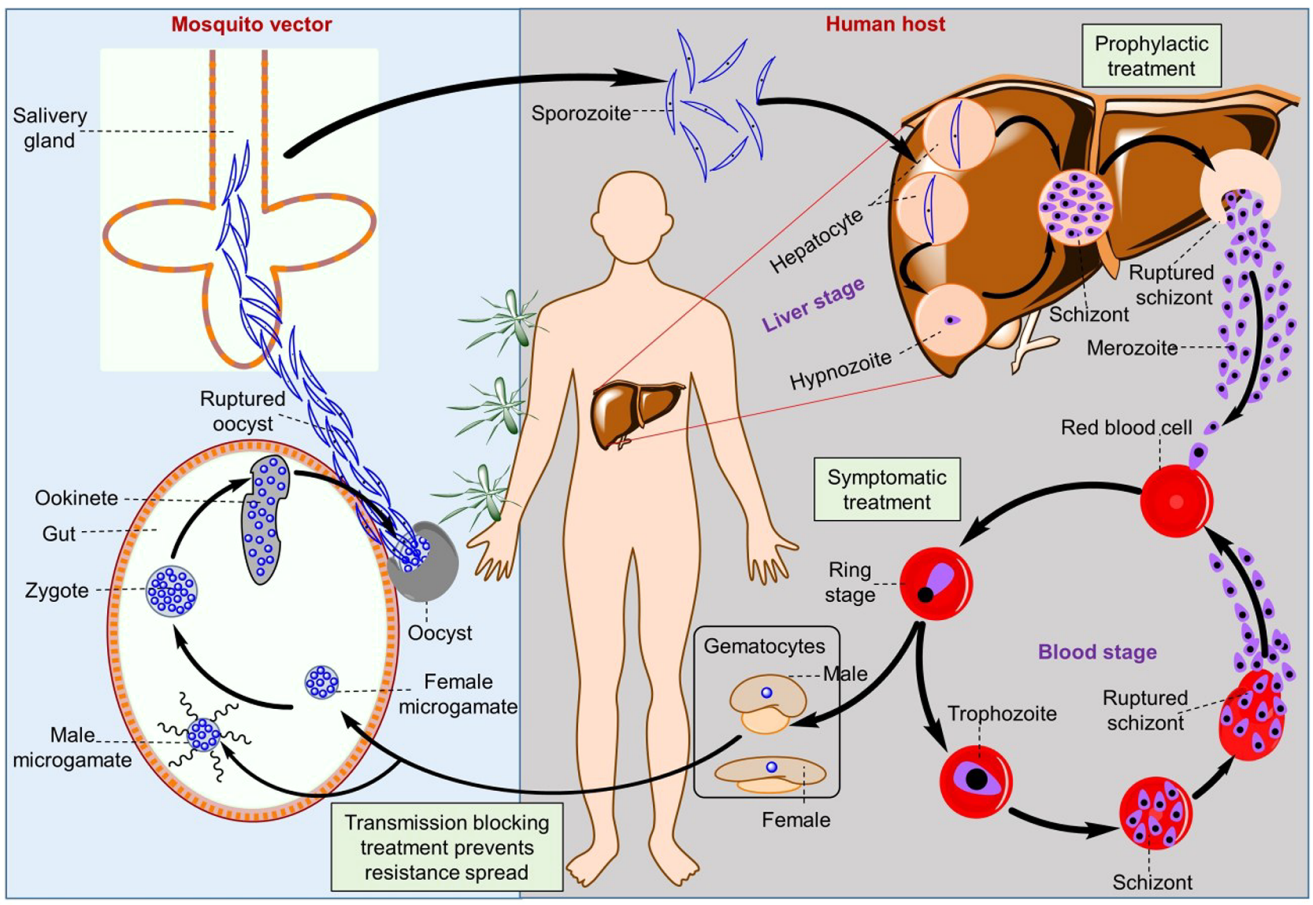
Schematic representation of the liver and blood stages of the *Plasmodium* spp. life cycle.

The primary treatment for malaria targets the disease-causing asexual blood stages (ABS) of *P. falciparum*, including various drug types such as 4-aminoquinolines (piperaquine and amodiaquine), antifolates (pyrimethamine and sulfadoxine), and endoperoxides (artemisinin and its derivatives artesunate, artemether, and dihydroartemisinin) (Figure 2).^10^ Artemisinin-based combination therapies (ACTs) are widely used as the first-choice treatment globally.^10^ In recent years, resistance to single and combination therapies, including ACTs, has emerged, leading researchers to focus on new chemotypes that are not susceptible to previous resistance mechanisms.^11,12^ Another strategy is to develop drugs that prevent malaria by targeting parasites in their early stage of development in the liver, before symptomatic blood-stage infection. The number of parasites in the early liver stages is relatively much lower in the liver versus the blood stage, reducing the likelihood of drug resistance and making liver-stage active compounds suitable for chemoprotection and mass drug administration.^10,13^ Furthermore, while the blood stage infection of *P. vivax* or *P. ovale* patients can be treated with blood schizonticides, the hypnozoites in the liver are unaffected by most antimalarials, thereby requiring treatment with 8-aminoquinolines drugs primaquine or tafenoquine in combination with chloroquine to achieve radical cure and prevent relapse.^14^ The 8-aminoquinolines are contraindicated in patients with glucose-6-phosphate dehydrogenase deficiency and cannot be administered during pregnancy, leading to an unmet clinical need.^15^

**Figure 2:**
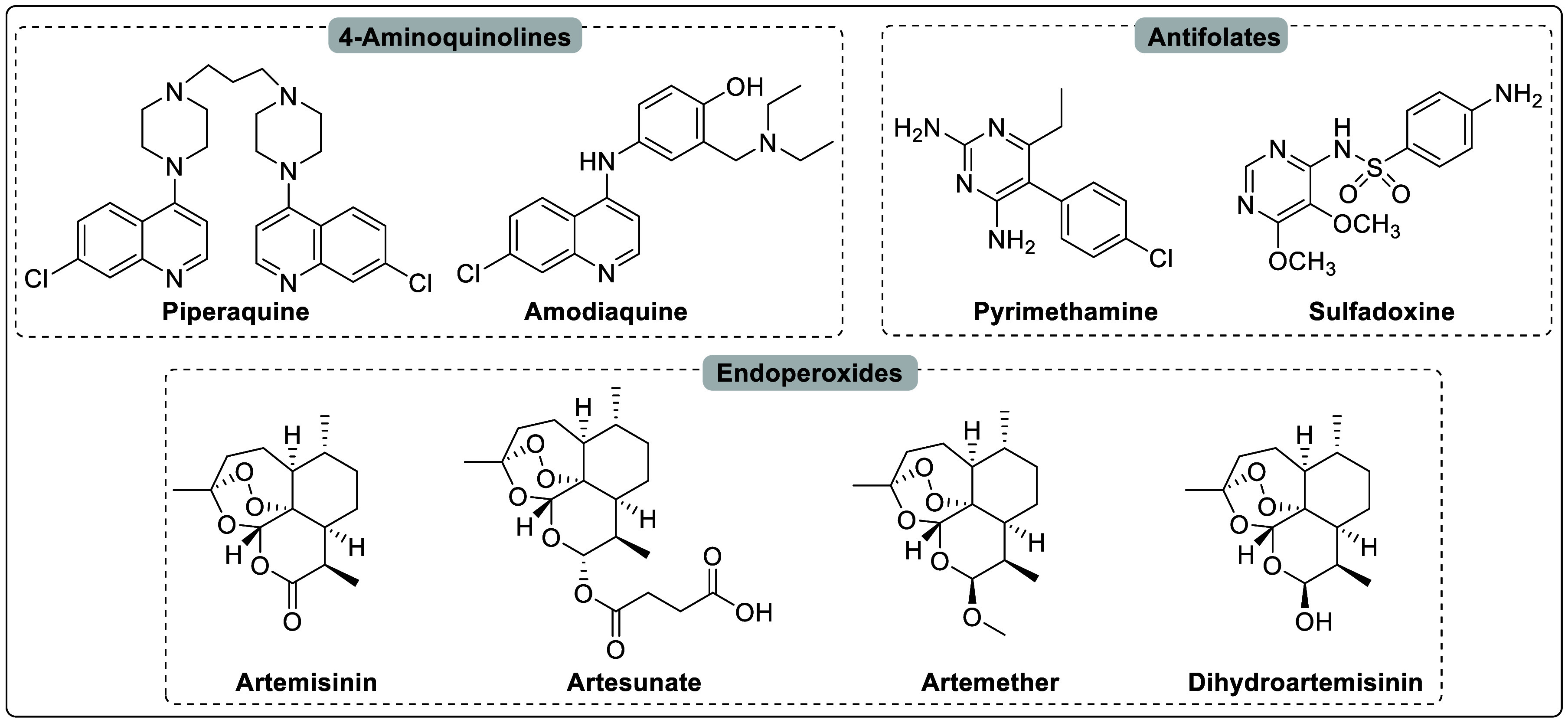
Chemical structures of the important classes of drugs that target the asexual blood stage of *P. falciparum*.

There is currently a great deal of interest in finding compounds that can target both liver and blood stages of *Plasmodium*, a species that causes malaria. Natural products and their analogs have historically played an important role in drug discovery for cancer and infectious diseases.^16−19^ Natural products possess various scaffold diversities and structural complexities, occupying biologically relevant chemical space with interesting properties.^20−22^ Artemisinin, a component of ACTs, is a natural product isolated from the plant *Artemisia annua* and has been a significant contribution to antimalarial drug discovery.^23^ Trichothecenes (TCNs), a large group of tricyclic sesquiterpenoid mycotoxins produced as secondary metabolites by various filamentous fungal species,^24,25^ are potential candidates for antimalarial drug discovery. TCNs exhibit a broad range of biological activities, such as antibacterial, antimalarial, antifungal, and antitumor.^26−28^ Among these, anguidine, a non-macrocyclic TCN isolated from the fungi of the genus *Fusarium*, has been reported to inhibit the initiation of protein synthesis.^29,30^ Anguidine is among the simplest natural products of the TCN family and is among the few TCNs whose total synthesis is reported.^26,31^ Verrucarin A,^32−34^ a macrocyclic TCN, and verrucarol,^35,36^ a non-macrocyclic TCN, are other examples of TCNs whose total syntheses are reported and also available commercially. Despite being commercially available, their multistage antimalarial activity against *Plasmodium* parasites has not been reported. Therefore, in this study, we evaluated the in vitro antimalarial activity of anguidine, verrucarin A, and verrucarol against the blood stages of *P. falciparum* and liver stages of *P. vivax*.

**Figure 3:**
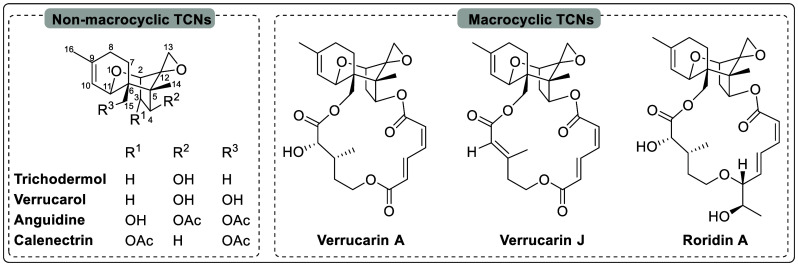
Chemical structures of the selected macrocyclic and non-macrocyclic TCNs.

## Results and Discussion

Antimalarial drug discovery most frequently begins with high-throughput, whole-cell phenotypic screening of *P. falciparum* ABS parasites.^37^ This is because this species and stage can be routinely cultured, allowing laboratory-adapted strains to be rapidly tested in automated drug discovery environments. This species and stage is also the largest contributor to malaria morbidity and mortality, making it the top priority for antimalarial targeting.^13^ In continuation of our work toward identifying antimalarial synthetic small molecules^38−40^ and natural products,^41−43^ we determined the potency of anguidine, verrucarin A, and verrucarol against the asexual blood stages of *P. falciparum*, with selectivity calculated against cytotoxicity to the human hepatoblastoma cell line HepG2 (Table 1). For this, we used a standard ABS assay in which ring stage parasites are treated and quantified after 72 h using high content imaging.^39^ Following three independent experiments, we found anguidine and verrucarin A were very potent against the ABS, with EC_50_’s of 6.16 and 0.45 nM, respectively, while verrucarol was nearly inactive at the highest concentration tested. Anguidine and verrucarin A were also found cytotoxic, leading to a very low selectivity index calculated ranging from 4.43 to 7.95.

**Table 1:** Potency of TCNs against *Plasmodium* spp.

compound	*P. falciparum* ABS (W2) EC_50_, nM (pEC_50_)	HepG2 Tox CC_50_, nM (pCC_50_)	ABS-HepG2 SI	*P. vivax* schizont^b^ EC_50_, nM (pEC_50_)	*P. vivax* hypnozoite EC_50_, nM (pEC_50_)	PHH Tox CC_50_, nM (pCC_50_)	schizont^b^-PHH SI	hypno-zoite -PHH SI
anguidine	6.16 (8.21 ± 0.06)	27.5 (7.56 ± 0.08)	4.43	13.5 (7.87 ± 0.55)	36.3 (7.44 ± 0.75)	380 (6.42 ± 0.31)	28.7	10.7
verrucarin A	0.45 (9.35 ± 0.04)	3.55 (8.45 ± 0.18)	7.95	4.07 (8.39 ± 0.82)	30.9 (7.51 ± 0.11)	316 (6.50 ± 0.05)	77.7	10.1
verrucarol	>4370 (<5.36 ± 0.20)	>10 000 (<5.00 ± 0.00)	N/A	>10 000 (<5.00 ± 0.00)	>3311 (<5.48 ± 0.68)	>10 000 (<5.00 ± 0.00)	N/A	N/A
dihydroart-emisinin^a^	1.44 (8.84 ± 0.09)							
puromycin^a^		398 (6.40 ± 0.10)		2290 (5.64 ± 0.47)	776 (6.11 ± 0.07)	1740 (5.76 ± 0.00)	<1.00	2.25
nigericin^a^				5.13 (8.29 ± 0.25)	12.3 (7.91 ± 0.25)	>200 (6.70 ± 0.00)	>38.9	>16.3

a Positive control.

b *P. vivax* liver schizonts; ABS (W2), asexual blood stage strain W2; HepG2 Tox, HepG2 cytotoxicity; SI, selectivity index; PHH Tox, primary human hepatocyte cytotoxicity. Shown are the geometric means of all EC_50_ values from all independent experiments, as well as the pEC_50_ or pCC_50_ values (i.e., the −log of EC_50_ or CC_50_ in [M]) including the standard deviation from all independent experiments in parentheses.

To further investigate the activity of these compounds, we next tested them against the liver stages of *P. vivax*, with selectivity calculated against cytotoxicity to the host primary human hepatocytes used for *P. vivax* liver stage assays.^44^ The *P. vivax* liver stage can be routinely tested in short-term assays (7–14 days), but a continuous in vitro culture of the blood or liver stages has not been established. Therefore, *P. vivax* liver stage assays are initiated using sporozoites generated by first collecting gametocyte-infected blood from malaria patient volunteers in endemic countries, feeding the blood to laboratory-reared mosquitoes, harvesting sporozoites, and infecting primary human hepatocyte cultures for potency assays. Because individual patient isolates cannot be cultured, they also cannot be cloned, expanded, or cryopreserved for repetitive use, meaning genetically distinct patient isolates are used for each experiment replicate. Furthermore, *P. vivax* liver stage parasites can be treated at different developmental points to obtain an activity profile that specifically translates to prophylactic versus radical cure outcomes. This is because once hypnozoites are established in the host hepatocyte, they require several days to become fully quiescent and, therefore, are not susceptible to most antimalarials.^45^ After two independent radical cure experiments with a different patient isolate used for each experiment, we found anguidine and verrucarin A to be very potent against both liver schizonts and hypnozoites, with EC_50_’s ranging from 36.31 to 4.07 nM, and verrucarol to be inactive at the highest concentration tested (Table 1). After quantifying cytotoxicity against the host hepatocytes, we again found low selectivity indices for schizonticidal activity, ranging from 28.66 to 77.74, and for hypnozonticidal activity, ranging around 10, for these two compounds (Table 1).

Out of the three TCNs that were tested, verrucarin A (a macrocyclic TCN), showed the highest potency against the ABS of *P. falciparum* and the liver stages of *P. vivax*. Anguidine (a non-macrocyclic TCN) followed closely behind, while verrucarol (an alkaline hydrolysate of verrucarin A) was completely inactive (Table 1). From these results, we can see that there is a connection between the structure and the activities of these three compounds. The presence of a free hydroxyl group at C-4 and C-15 (as in verrucarol) led to a complete loss of activity. The presence or absence of a macrocycle at C-4 and C-15, as seen in verrucarin A and anguidine, was found to have no significant impact on the activity, toxicity, or selectivity of these chemical compounds. This suggests that the macrocycle’s presence or absence at these positions does not play a significant role in determining the aforementioned factors.

While we found anguidine and verrucarin A potent against *P. falciparum* ABS and *P. vivax* liver stage parasites, we also found high cytotoxicity and therefore very low selectivity. These findings raise the question of whether or not these compounds, which are known to be broadly cytotoxic protein synthesis inhibitors, are acting on any parasite synthesis processes directly or if instead the effect is on the host cell (either erythrocyte or primary hepatocyte), leading to indirect effects on the intracellular parasite. Multiple pieces of data suggest that the effect is directly on the parasite. First, erythrocytes are thought to undergo little to no mRNA translation, thus activity on ABS parasites would be directly on the parasite’s protein synthesis and indicative of a direct killing effect.^46^ Second, in the liver stage, both the host hepatocyte and parasite (either schizont or hypnozoite) perform protein synthesis, but despite low selectivity, we saw a small selectivity window that was greater than that of another protein synthesis inhibitor, puromycin (Table 1). These data suggest that liver stage parasite killing from some broadly active compounds, like puromycin, is an indirect effect of a dying host cell environment, while the same parasites are more sensitive than their host cells to our broadly active TCNs, which also indicates a direct effect.

Ultimately, these compounds are much too toxic for drug development; so much so that during the course of these studies anguidine was listed as a U.S. Department of Health and Human Services Select Agent.^47^ Despite the low selectivity, hits against mature hypnozoites (i.e., those tested in a radical cure assay format) are incredibly rare, as these forms are relatively metabolically silent as they quietly persist in tissue for months or years before reactivating.^44^ Because anguidine and verrucarin A do show a selective effect on hypnozoites, this suggests hypnozoites are indeed performing some protein synthesis, which is consistent with a recent report showing evidence that hypnozoites maintain proteasome activity and are therefore undergoing protein turnover.^48^ As such, these compounds could be utilized as chemical probes in line with a recent report in which a modified form of puromycin was used to quantify protein synthesis as an end point in a small-molecule screen for protein synthesis inhibitors.^49^

## Conclusion

We conducted a study to test the effectiveness of commercially available TCNs (anguidine, verrucarin A, and verrucarol) against the asexual blood stages of *P. falciparum* and liver stages of *P. vivax*. The results showed that anguidine and verrucarin A were highly effective against both the blood and liver stages of *Plasmodium* parasites. However, verrucarol, which contains free hydroxyl groups at C-4 and C-15 positions, was found to be completely inactive at the highest concentration tested. It was observed that anguidine and verrucarin A had high cytotoxicity and low selectivity, indicating that these compounds could be directly affecting parasite synthesis processes or indirectly affecting intracellular parasites via the host cell. However, further studies revealed that the effect was indeed directly on the parasite. Although the anguidine and verrucarin A showed low selectivity for ABS of *P. falciparum* and the liver stage of *P. vivax* schizonts, these active compounds showed some selectivity on the liver stage of *P. vivax* hypnozoites. This suggests that hypnozoites are indeed performing some protein synthesis. Overall, due to their low selectivity, these active compounds may not be suitable for drug development. However, they could be utilized as chemical probes.

## Experimental Section

### General Information

All reagents and solvents were obtained from Fischer Scientific, Sigma-Aldrich, or TCI America and used without further purification. Anguidine, also known as diacetoxyscirpenol (catalog number: 34137) was purchased from Sigma-Aldrich as a diacetoxyscirpenol solution in acetonitrile (100 μg/mL), analytical standard (HPLC purity ≥98%). Verrucarin A (catalog number: V4877, HPLC purity ≥95%) and verrucarol (catalog number: V1628, TLC purity ≥99%) were purchased as a powder from Sigma-Aldrich.

### General Experimental Procedures

**Figure 4:**
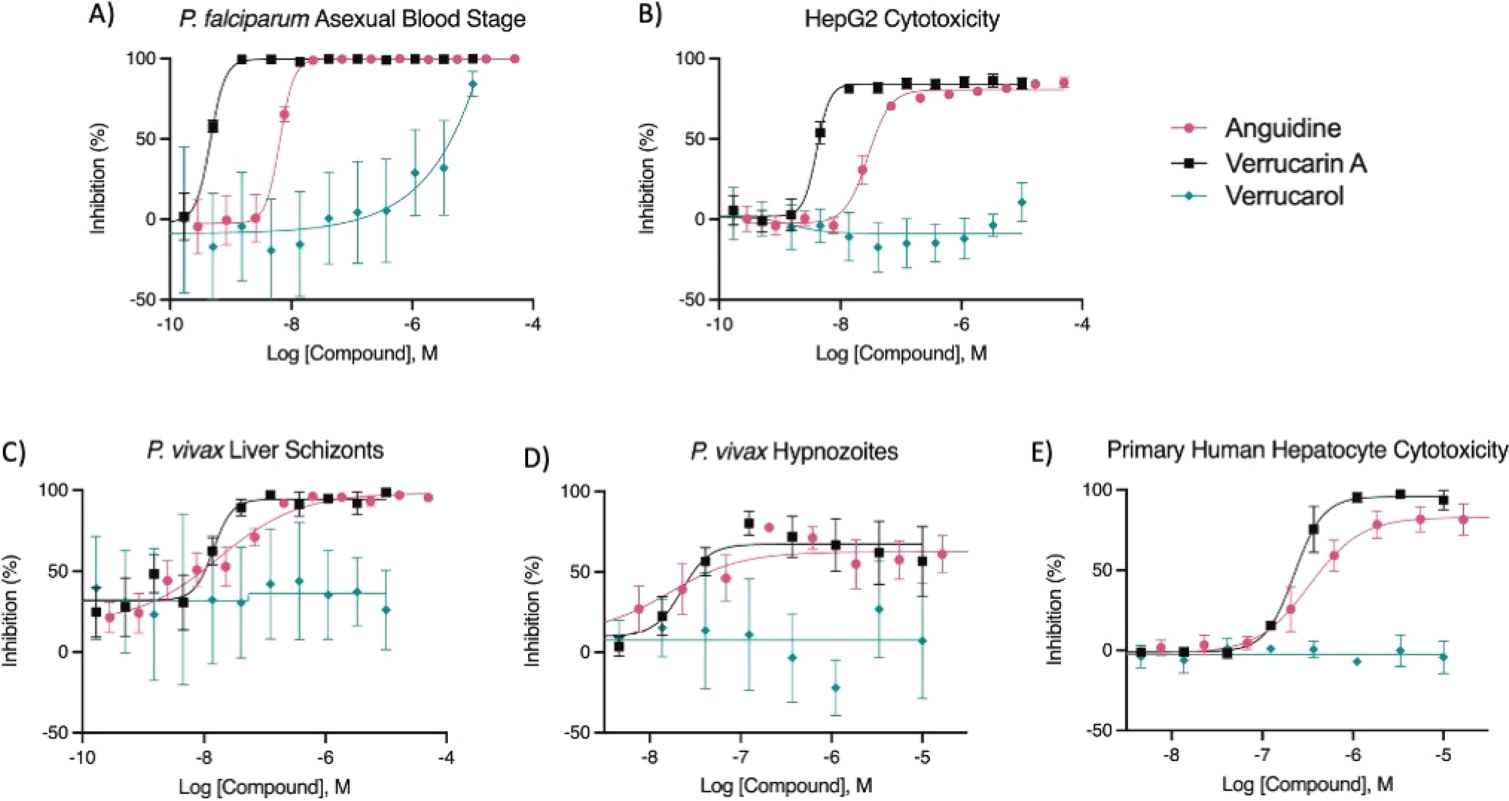
Dose-responsive inhibition studies of anguidine, verrucarin A, and verrucarol against (A) *P. falciparum* asexual blood stage, (B) HepG2 cytotoxicity, (C) *P. vivax* liver schizonts, (D) *P. vivax* hypnozoites, and (E) primary human hepatocyte cytotoxicity. Bars represent SEM from duplicate technical replicate wells pooled from three (a, b) or two (c–e) independent experiments.

All source compound plates were prepared by making a 1000× stock of test compound in DMSO (i.e., 10 or 50 mM), spotting 15 μL of compound into 384-well conical-bottom microtiter plates (Greiner Bio-one), and using a Beckman 4000 to make a 12-point, 1:3 semilog dilution series as previously described.^39,50^ All raw high-content imaging data from *P. falciparum* blood stage, HepG2, and *P. vivax* liver stage assays were loaded into the CDD Vault for normalization and curve fitting. The positive controls for the *P. vivax* liver stage, the *P. falciparum* blood stage, and HepG2 assays were nigericin, artemisinin, and puromycin, respectively, while 0.1% v/v DMSO-treated wells served as the negative control for all assays. pEC_50_ and pCC_50_ values (the −log of the EC_50_ or CC_50_ in [M]) were calculated from individual runs; Table 1 shows the average and standard deviation of values from all independent experiments. The curves displayed in Figure 4 were generated in Prism (Graphpad) from normalized values from duplicate wells from each independent experiment and pooled together to obtain one curve for each assay and test compound.

### *P. falciparum* Asexual Blood Stage Experiments Were Performed as Previously Described^39^

Briefly, assays were initiated by plating 40 μL of ring-stage *P. falciparum* (strain W2) asexual blood stage parasites at 2% parasitemia and 0.75% hematocrit in 384-well plates (Greiner Bio-one). The red cells were allowed to settle before addition of 40 nL of 1000× compound from a source plate (see above) using a pin tool affixed to a Beckman Coulter NXp. After 72 h, cultures were fixed, stained with Hoechst 33342 for DNA content, and quantified using an ImageXpress high content imager (Molecular Devices).

### HepG2 Cytotoxicity Experiments Were Performed as Previously Described^39^

Briefly, assays were initiated by plating 40 μL of culture media containing 2000 HepG2 cells (catalog HC-8065, ATCC) into collagen-coated 384-well microtiter plates. The following day, cultures were treated by addition of 40 nL of 1000× compound (from above) using a pin tool affixed to a Beckman Coulter NX^p^. After 72 h, cultures were fixed, stained with Hoechst 33342 for DNA content, and quantified using an ImageXpress high-content imager (Molecular Devices).

The full, step-by-step protocol for rearing mosquitoes, feeding a *P. vivax-*infectious blood meal to mosquitoes, dissecting mosquitoes, seeding assay plates with primary human hepatocytes, infecting cultures, treating cultures, and quantifying liver stage growth and host hepatocyte cytotoxicity is published.^50^ The Cambodian human subject protocols for this study were approved by the Cambodian National Ethics Committee for Health Research (104NECHR and 094NECHR). Protocols conformed to the Helsinki Declaration on Ethical Principles for Medical Research Involving Human Subjects,^51^ and informed written consent was obtained for all volunteers or legal guardians. Briefly, assays were initiated by seeding 18 000 primary human hepatocytes (lot BGW, BioIVT) into each well of a 384-well, collagen-coated microtiter plate. Two days after seeding, mosquitoes that had been fed *P. vivax-*infected blood 18 days prior were dissected to obtain sporozoite-laden salivary glands. Glands were crushed to release sporozoites, which were added to culture wells. From days 5–7 postinfection, mature hypnozoites and growing schizonts were treated with test compound,^44^ as per the radical cure assay mode.^50^ At 12 days postinfection, cultures were fixed, stained with immunofluorescence reagents against *Pv*UIS4,^52^ and imaged on a Lionheart high-content imager (Agilent).
